# Translating Evidence‐Based Self‐Management Interventions Using a Stepped‐Care Approach for Patients With Cancer and Their Caregivers: A Pilot Sequential Multiple Assignment Randomized Trial Design

**DOI:** 10.1002/pon.70043

**Published:** 2025-01-06

**Authors:** Sylvie Lambert, Erica E. M. Moodie, Jane McCusker, Marion Lokhorst, Cheryl Harris, Tori Langmuir, Eric Belzile, Andrea Maria Laizner, Lydia Ould Brahim, Sydney Wasserman, Sarah Chehayeb, Michael Vickers, Lindsay Duncan, Mary Jane Esplen, Christine Maheu, Doris Howell, Manon de Raad

**Affiliations:** ^1^ Ingram School of Nursing McGill University Montreal Canada; ^2^ St. Mary's Research Centre Montreal Canada; ^3^ Department of Epidemiology, Biostatistics, and Occupational Health McGill University Montreal Canada; ^4^ Université de Montréal Institut Universitaire en Santé Mentale Douglas Montreal Canada; ^5^ The Ottawa Hospital Research Institute Ottawa Canada; ^6^ Department of Psychology, Social Sciences University of Ottawa Ottawa Canada; ^7^ Concordia University Montreal Montreal Canada; ^8^ Research Institute McGill University Health Centre Montreal Canada; ^9^ The Ottawa Hospital Ottawa Canada; ^10^ Department of Kinesiology and Physical Education McGill University Montreal Canada; ^11^ Department of Psychiatry Temerty Faculty of Medicine University of Toronto Toronto Canada; ^12^ Department of Supportive Care Princess Margaret Cancer Centre Toronto Canada

**Keywords:** caregivers, neoplasms, psycho‐oncology, psychosocial intervention, self‐management

## Abstract

**Background:**

Self‐directed interventions are cost‐effective for patients with cancer and their family caregivers, but barriers to use can compromise adherence and efficacy.

**Aim:**

Pilot a Sequential Multiple Assignment Randomized Trial (SMART) to develop a time‐varying dyadic self‐management intervention that follows a stepped‐care approach in providing different types of guidance to optimize the delivery of Coping‐Together, a dyadic self‐directed self‐management intervention.

**Methods:**

48 patients with cancer and their caregivers were randomized in Stage 1 to: (a) Coping‐Together (included a workbook and 6 booklets) or (b) Coping‐Together + lay telephone guidance. At 6 weeks, change in distress level was assessed, and non‐responding dyads were re‐randomized in Stage 2 to (a) continue with their Stage 1 intervention or (b) be stepped‐up. Benchmarks for acceptability, feasibility, and clinical significance (anxiety and quality of life (QOL)) were assessed via surveys and study logs.

**Results:**

Feasibility was supported by a low refusal rate at ≤ 30% and < 10% missing data. Men and women were enrolled in at least a 40:60 ratio for caregivers, but less for patients. Recruitment was slow at 1 dyad/week. Acceptability was supported by a low attrition rate (12.5%) and with 87% of participants finding the booklets helpful. Telephone guidance in Stage 1 increased adherence to Coping‐Together; however, in Stage 1, participants benefited more from the self‐directed format than the guidance. All patients who were stepped‐up in Stage 2 benefited from their new assignment; this trend was less clear for caregivers.

**Significance:**

Findings suggest a 3‐step approach to dyadic self‐management support that warrants further testing.

**Trial Registration:**

Clinical Trials Registration #: NCT04255030.

## 1 Introduction

On average, 90% of patients experience 8–9 burdensome physical symptoms and/or psychosocial concerns from cancer and/or its treatment [1]. As treatment is increasingly delivered in ambulatory clinics, once at home, all patients bear the brunt of responsibility for self‐managing their cancer challenges. This, no matter their readiness to do so, at varying levels of success, and mostly without direct supervision from clinicians [2]. Cancer challenges are not self‐managed in a vacuum; family caregivers are often relied on [3]. Self‐managing cancer challenges for patient‐caregiver dyads requires that they develop skills (e.g., communication), improve their self‐efficacy, and access resources [3, 4]. However, patients and, even more so caregivers, often do not receive the training needed for effective self‐management [5]. Consequently, they commonly undertake self‐directed learning using online resources, but the self‐management content of these resources is often of low quality [6, 7].

Despite the efficacy of many self‐management support interventions for patients [8] and caregivers [3], these are rarely implemented in usual care [2]. Mostly, this is because self‐management trials rely on high‐intensity formats, that is, multiple, in‐person sessions delivered by a dedicated team. This format is also burdensome (e.g., travel) for patients and caregivers, with attrition as high as 50% [9]. We also know that high‐intensity interventions are needed by less than 15% of patients [10]. Thus, opportunities exist to mitigate the impact of cancer for a large proportion of patients/caregivers using less intense formats.

This motivated the development of Coping‐Together, a one‐stop shop for tailored self‐management support for patient‐caregiver dyads [11]. As a workbook‐ and booklet‐based self‐management repository for most cancer challenges, dyads pick and choose what they want to learn to self‐manage. Coping‐Together's mechanisms of actions are mainly explained by the Stress and Coping Framework [12]. In brief, Coping‐Together helps patients and caregivers understand cancer's challenges, expand their repertoire of individual and dyadic coping strategies, and improve their self‐efficacy in implementing these to manage key challenges in a way that foster better health outcomes (see Figure S1: Supporting Information S1 for mechanism of action in supplementary material for details). Acceptability and pilot studies found Coping‐Together can increase dyads' active coping [11, 13]. However, some dyads needed more help navigating the resource.

Stepped‐care can optimize interventions in a cost‐effective manner [14]. In Step 1, a low‐intensity intervention, like Coping‐Together, is often offered. For many patients, Step 1 will meet their needs, but for those needing more support, in Step 2 more clinician contact is usually offered [14]. However, we have found in two randomized controlled trials that guidance from a trained, non‐health care professional (lay) over the phone enhanced adherence to and efficacy of a paper‐based, self‐directed depression toolkit among primary care patients [15] and cancer survivors [16]. These studies did not examine adding clinician guidance for those who did not benefit from lay guidance. In addition to shown efficacy, lay guides were chosen because many cancer centers have volunteers who could provide this service, if eventually implemented in clinical practice.

The present pilot study evaluated the feasibility, acceptability, and clinical significance (on anxiety and quality of life (QoL)) of adding lay or clinician guidance to Coping‐Together to optimize its efficacy using a stepped‐care approach. Table 1 details the a priori benchmarks for the present pilot (based on prior research [9, 11, 13, 18, 19]) that needed to be attained to justify a larger trial.

**TABLE 1:** Overview of a priori benchmarks for the present pilot and results.

Criteria		Benchmarks	Results
Feasibility	Recruitment	2–3 dyads/week	1 *recruitment amidst COVID
	Refusal rate	≤ 30%	30.2%
	Missing data	< 10%	Baseline < 3%
			Follow‐up < 7%
	Men: Women	40:60	31:69 patients, 46:54 caregivers
Acceptability	Attrition	< 20%	12.5%
	Helpfulness	≥ 75%	Workbook: Coping‐together + lay guidance = 89%, self‐directed coping‐together = 63%
			Booklets: Coping‐together + lay guidance = 100%, self‐directed coping‐together = 78%
	Adherence self‐directed	75% new self‐manage skill	Coping‐together + lay guidance = 100%; self‐directed coping‐together = 58%
	Adherence guidance	75% high adherence	73.9% for lay guidance
			100% for MI
Clinical significance	Effect size	0.2 (at least a small effect size [17])	Anxiety ‐ stage 1 pooled ES = −0.25; stage 2 pooled ESs < 0.2
			QOL—Stage 2 responders patients < 0.2, caregivers −0.36 and stage 2 non‐responders patients 0.54, caregivers < 0.2
	Minimal clinically important difference (MCID)	25% of participants improve on the primary outcomes by at least the MCID	In Stage 1, more patients and caregivers improved by the MCID on anxiety in the self‐directed Coping‐Together
			In Stage 2, for patients, more improved by the MCID on anxiety and QOL in the lay guidance group. For caregivers, higher improvements remained in the self‐directed group

## 2 Methods

**FIGURE 1:**
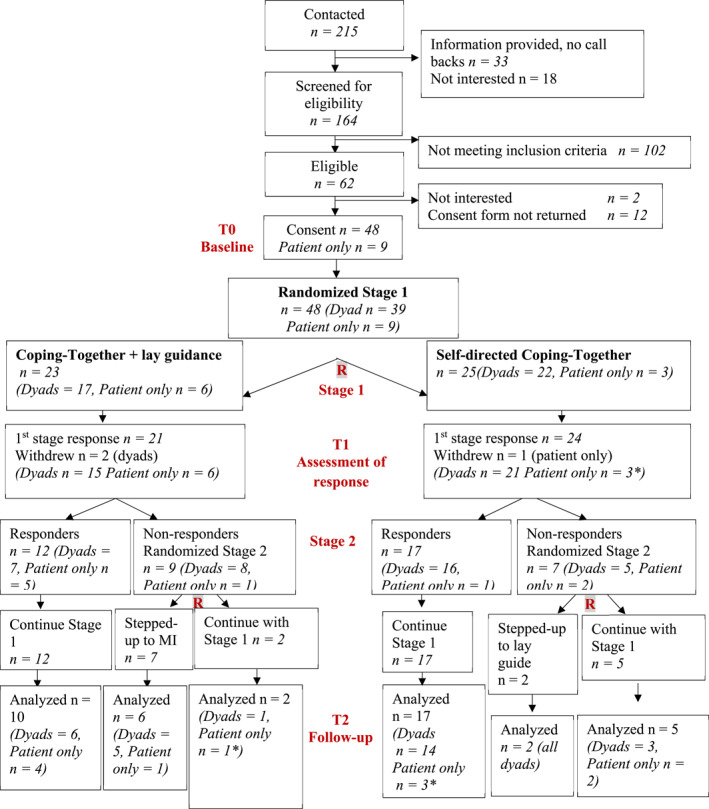
SMART design and Flow diagram. *Caregiver dropped out, leading to fewer dyads and additional patient only.

A multi‐centre, pilot Sequential Multiple Assignment Randomized Trial (SMART) was conducted. SMARTs are characterized by different interventions compared at different time points to determine what is optimal, for whom, and when [20]. This study was guided by the CONSORT guidelines [21]. Figure 1 details the design. All procedures were performed in compliance with relevant laws and institutional guidelines and a multicentre ethics approval was obtained (REB number: 17‐10, 2017).

### 2.1 Sample

Patients were diagnosed with a Stage 0‐III cancer, with a particular focus on breast, prostate, or colorectal cancer; they received treatment (including surgery, radiotherapy, chemotherapy, and/or hormone therapy, but excluding active surveillance) in the last 12 months or scheduled to receive treatment imminently; and they identified a caregiver (friend or spouse, partner, or other family member) willing to participate. Toward the end of the pilot, some patients enrolled alone because they expressed a need for the intervention, but their caregiver was not available. We tested the acceptability of allowing one member of the dyad to enroll independently, anticipating this situation might often occur in real‐world settings. To avoid a floor effect, the patient or caregiver had to report at least low anxiety at recruitment (i.e., Distress Thermometer (DT) [22] score ≥ 4). Patients/caregivers needed access to a computer with internet and e‐mail capabilities and to understand English. Caregivers receiving treatment for cancer were excluded. If the patient/caregiver was hospitalized, had suicidal intent, received psychological treatment, had recently participated in a similar program, or had moderate‐severe cognitive impairment, the dyad was excluded.

### 2.2 Sample Size

Based on Almirall et al.‘s algorithm [23], the target sample size was 44 dyads, setting the probability of having 4 dyads/each Stage 2 interventions at 90% and the response rate to Stage 1 interventions at 40%.

### 2.3 Recruitment

Recruitment occurred at three cancer centers in Canada in 2021–2022, whereby clinicians introduced the study to patients during scheduled appointments or sent an invitation letter to those without an appointment during this time. Clinic posters/pamphlets invited self‐referral as well as dissemination of the study through social media, email lists, newsletters, and websites of community organizations. Invitation letters were also sent to patients who participated in previous studies by the team (but had not been exposed to Coping‐Together previously) and, at one center, through a patient portal. The final sample was all recruited from the cancer centers. A research assistant (RA) completed eligibility interviews with patients and then caregivers. Those eligible were directed to an online consent form and baseline survey.

### 2.4 Randomization

A real‐time [23], computer‐generated randomization schedule was created to randomize dyads on a 1:1 basis, using random block sizes of two or 4. Randomization was stratified by severity of anxiety (highest score within the dyad on the baseline Hospital Anxiety and Depression Scale (HADS)‐Anxiety subscale [24]). The randomization schedule was programmed into a secure randomization service, accessible only to the study coordinator and ensured allocation concealment. Stage 1 non‐responders were (re)randomized to Stage 2 using a similar approach.

### 2.5 Stage 1 and 2 Self‐Management Interventions

Coping‐Together's delivery varied over time following a stepped‐care approach (see Figure 1). All participants continued with usual care. Two of the three cancer centers offered psychosocial services via oncology team referrals, with one center incorporating distress screening in clinical practice. Participants were not blinded to group allocation; they were blinded to the study objectives.

#### 2.5.1 Stage 1

Dyads were randomized to intervention (a) or (b):

- *Self‐directed Coping‐Together*. Dyads used the Coping‐Together workbook and booklets for 6 weeks, in a self‐directed manner (available at https://www.mcgill.ca/self‐management/coping‐together‐booklets). The booklets addressed challenges of (a) symptom management, (b) coping with anxiety, (c) collaborating with the health care team, (d) engaging in shared decision‐making, (e) communicating with partner/family, and/or (f) obtaining community resources. A relaxation CD was also included. The workbook introduced each booklet and included a checklist of challenges, allowing participants to select which ones they want to address. The workbook then guides participants in choosing and learning coping strategies for their challenges (e.g., goal setting, action planning).
- *Coping‐Together + Lay Guidance*. Dyads received, in addition to Coping‐Together, support via six weekly telephone calls (15–20 min) from a lay guide. Guides are not therapists; they focus on (a) introducing Coping‐Together; (b) supporting the identification of a challenge they want to learn to self‐manage and create a coping plan; and (c) encourage adherence by setting a SMARTTER goal (Specific, Measurable, Attainable, Relevant, Time‐Oriented, TogethEr and Rewards). Call #1 oriented dyads to Coping‐Together and Call #2 explored its initial use. Calls #3 to 6 focused on guiding dyads through their coping plan by setting a SMARTTER goal. At each call, guides explored dyads' use of Coping‐Together and tailored their approach. Three guides were trained with the help of a certified couple's therapist based on a manual that included a script for each call. Guides were supervised by the study coordinator who reviewed recorded calls to provide ongoing feedback.

#### 2.5.2 Response Screening

After 6 weeks, the DT was re‐administered online to both the patient and caregiver to determine response to Stage 1 intervention (primary tailoring variable). If both member of the dyads had a DT score ≥ 5 at screening, their DT scores needed to decrease by at least one point between screening and the decision point for the dyad to be a responder. If only one member of the dyad had a DT score ≥ 5 at screening, their score needed to decrease by one point and the other member's score had to not increase by the MCID (by one point if score at recruitment was ≥ 5, by two points if recruitment score < 5) for the dyad to be responder.

#### 2.5.3 Stage 2

Responders to Coping‐Together continued using it without specific instructions and those who responded to the lay guidance, received three maintenance calls in Stage 2. However, non‐responders to Stage 1 self‐directed Coping‐Together were (re)randomized to continue with their Stage 1 assignment, without additional instructions or stepped‐up to Coping‐Together + Lay Guidance, as previously described.

Whereas non‐responders who had already received lay guidance in Stage 1 were (re)randomized to continue with lay guidance (three more calls) or stepped‐up in Stage 2 to Coping‐Together + Lay Guidance + motivational interviewing (MI). Details of the differences between lay guidance and MI are included in Table S2: in Supporting Information S1. The goal of the MI was to strengthen motivation/confidence for adopting self‐management skills. These dyads received six, 30‐min weekly telephone‐based sessions by a mental health nurse. The first call included a handover from the lay guide to the MI. Calls focused on (a) identifying dyads' concerns, (b) reviewing self‐management efforts, (c) identifying goals, (d) identifying skills needed to achieve goals, and (e) addressing barriers to self‐management. At each call, progress, goals, and corresponding plans were discussed.

### 2.6 Data Collection

#### 2.6.1 Surveys

Patients and caregivers completed a self‐administered, clinical significance outcomes survey at baseline and a follow‐up survey at the completion of Stage 2 (∼12 weeks post‐baseline). The baseline survey included demographic questions and all primary and secondary measures summarized in Table 2. The follow‐up survey included the baseline measures plus questions pertaining to usual care and co‐interventions, any changes in diagnosis/treatment, and Coping‐Together use items [11].

**TABLE 2:** Primary and secondary clinical significance outcomes.

Outcomes	Measures	
	Patients	Caregivers
Primary		
Anxiety	7‐Item hospital and anxiety depression scale (HADS)‐anxiety [24] (α = 0.68–0.93) [25]	
Quality of life	27‐Item functional assessment of cancer therapy‐general [26] (FACT‐G α > 0.75)	35‐Item quality of life index‐cancer (α = 0.91) [27]
Secondary		
Depression	7‐Item HADS‐depression [24] (α = 0.68–0.93) [25]	
Coping	37‐Item dyadic coping inventory [28] (α = 0.63–0.84) captured how partners support one another	
	28‐Item brief COPE [29] (individual coping strategies)	
Appraisal	10‐Item perceived stress scale [30], α = 0.73–0.91, measures appraisal of a situation as stressful	
	28‐Item cognitive appraisal of health scale captures appraisal of illness as a threat, benign/irrelevant, a harm/loss, and/or a challenge (α > 0.70) [31]	
Self‐management skills	Health education impact questionnaire [32] (α = 0.70) measures active engagement, skills, constructive attitude, self‐monitoring, health services navigation, social integration, health directed activity, and distress	
	Health literacy questionnaire (α = 0.76–0.94) [33] subscales: Having sufficient information to manage my health, actively managing my health, and ability to find good health information	
Dyadic adjustment	Revised dyadic adjustment scale [34] (consensus, satisfaction, cohesion, and affective expression)	

#### 2.6.2 Study Log

The following information was collected: number of participants referred/self‐referred, number of individuals eligible/ineligible, number of eligible individuals who declined participation, and number of people who withdrew.

#### 2.6.3 Fidelity Guidance Calls

Lay guidance calls were logged and audio‐recorded, with a random sample of 25 calls then selected for fidelity monitoring. The fidelity checklist included items for content and procedures.

### 2.7 Data Analysis

Analyses were carried out in SAS 9.4 and STATA 17.0. From study log data, recruitment, refusal, and attrition rates were calculated. Descriptive statistics for sample characteristics were tabulated. The percentage of missing survey data was calculated. Adherence to Coping‐Together was calculated as the proportion of participants who applied a new self‐management skill. Adherence to the coach and MI calls were categorized as: low (< 25% of calls completed), moderate (25%–50%), or high (> 50%). The fidelity of guides' calls was determined on the proportion of items selected on the checklist. For the primary outcomes, groups were compared using effect sizes (ESs), calculated by computing differences between two estimated means divided by the pooled standard deviation. Adjusted analysis was conducted using linear regression; a model including the study group and the baseline score was fitted to estimate the ES of the intervention group. Generalized Estimation Equations were used to account for the correlation due to the clustering of patients and caregivers. The proportions of patients and caregivers who improved by the MCID of 1.5 points on the HADS‐Anxiety [35] and patients who improved by three points [36] on the FACT‐G were calculated (see Table 2 for details on measures).

## 3 Results

Table 1 summarizes the results along the benchmarks.

### 3.1 Feasibility of Recruitment

Figure 1 details participants' flow. Overall, 39 dyads and 9 patients alone were randomized over 45 weeks; approximately 1 dyad/week. Top common reasons for non‐eligibility were cancer stage (*n* = 34, 34%) and no distress (*n* = 23, 23%). Out of 39 dyads, both patient and caregiver met the distress eligibility criterion in 13 dyads, only the patient in 19, and only the caregiver in 7 dyads. The refusal rate was 30.2%, this decreased to 14.9%, if we exclude those given information with no follow‐up (*n* = 33), as it is unknown how many were eligible.

### 3.2 Demographics

Table 3 provides a description of the sociodemographic characteristics. Three‐quarters of caregivers were patients' spouse/partner. Patients' and caregivers' mean age was 69.9 (SD = 10.8) and 66.6 (SD = 11.4) years, respectively. Males and females were reached in a proportion of 31:69 for patients and 46:54 for caregivers.

**TABLE 3:** Baseline demographic characteristics by stage 1 randomization group (*n* = 48).

Characteristics	Patients		Caregivers	
	Lay guidance (*n* = 23)	Coping‐together (*n* = 25)	Lay guidance (*n* = 17)	Coping‐together (*n* = 22)
	*n* (%)	*n* (%)	*n* (%)	*n* (%)
Dyads	17 (73.9)	22 (88.0)		
Relationship (among dyad = Yes)				
Spouse/partner	13 (76.5)	17 (77.3)		
Other	4 (23.5)	5 (22.7)		
Living with patient (among dyad = Yes)	14 (82.4)	16 (72.7)		
Age, mean (SD)	66.5 (10.1)	67.2 (11.6)	65.3 (10.4)	67.6 (12.3)
Sex				
Male	6 (26.1)	9 (36.0)	10 (58.8)	8 (36.4)
Female	17 (73.9)	16 (64.0)	7 (41.2)	14 (63.6)
Marital status				
Married/common law	14 (60.8)	20 (80.0)		
Other	5 (33.3)	5 (20.0)		
Education				
High school or below	2 (8.7)	2 (8.3)	2 (11.8)	3 (13.6)
Post secondary diploma	9 (39.1)	8 (33.3)	8 (47.1)	4 (18.2)
Undergraduate university	5 (21.7)	2 (8.3)	5 (29.4)	9 (40.9)
Graduate diploma	7 (30.4)	12 (50.0)	2 (11.8)	6 (27.3)
Employment				
Full time	8 (34.8)	4 (17.4)		
Part time	2 (8.7)	1 (4.4)		
Retired	13 (56.5)	16 (69.6)		
Other	0 (0.0)	2 (8.8)		
Income				
< 60,000	5 (21.7)	6 (24.0)	1 (5.9)	7 (31.8)
60,000–99,000	7 (30.4)	6 (24.0)	6 (35.3)	4 (18.2)
≥ 100,000	5 (21.7)	7 (28.0)	5 (29.4)	7 (31.8)
Prefer not to answer	6 (26.1)	6 (24.0)	5 (29.4)	4 (18.2)
Cancer*				
Breast	14 (60.9)	12 (50.0)		
Prostate	2 (8.7)	7 (29.2)		
Colorectal	4 (17.4)	2 (8.3)		
Other	8 (34.8)	5 (20.8)		
Stage				
0	2 (9.1)	1 (4.3)		
I	3 (13.6)	9 (39.1)		
II	13 (59.1)	5 (21.7)		
III	4 (18.2)	9 (39.1)		
Time since diagnosis				
< 6 months	5 (21.7)	7 (28.0)		
6–12 months	10 (43.5)	6 (24.0)		
> 12 months	8 (34.8)	12 (48.0)		
Treatment*				
Surgery	13 (56.5)	16 (64.0)		
Radiation	9 (39.1)	10 (40.0)		
Chemotherapy	12 (52.2)	9 (36.0)		
Other	7 (30.4)	9 (36.0)		
Mean number comorbidities (SD)	2.5 (2.1)	1.5 (1.2)	2.5 (2.8)	1.5 (1.2)
Distress thermometer score, mean (SD)	6.4 (1.3)	5.3 (2.3)	4.4 (2.4)	4.6 (1.9)
Hospital anxiety and depression—Anxiety subscale, mean (SD)	8.1 (4.0)	6.4 (4.4)	7.8 (4.4)	7.3 (3.5)
0–7	12 (52.2)	21 (84.0)	8 (47.1)	11 (52.4)
8–10	4 (17.4)	3 (12.0)	5 (29.4)	7 (33.3)
> 11	7 (30.4)	5 (20.0)	4 (23.5)	3 (14.3)
Hospital anxiety and depression—Depression subscale, mean (SD)	6.3 (3.2)	5.0 (3.2)	4.8 (3.5)	3.8 (3.0)
0–7	14 (60.9)	21 (84.0)	12 (70.6)	18 (85.7)
8–10	6 (26.1)	3 (12.0)	4 (23.5)	3 (14.3)
> 11	3 (13.0)	1 (4.0)	1 (5.9)	0

*Note:* * = some have more than 1 cancer or treatment.

### 3.3 Attrition

Attrition was low at 12.5% (See Figure 1). Attrition was higher in the lay guidance (21.7%) than the self‐directed (8%) group, but similar among responders (6.9%) and non‐responders (6.3%) in Stage 2. The main reasons for withdrawing from the study were already coping well, too busy, hospitalized or recovering post‐surgery, and difficulty using English booklets.

### 3.4 Adherence to Coping‐Together

Coping‐Together was used to identify a cancer challenge by similar proportions of patients (82.9%) and caregivers (78.6%). However, this was more frequently done in the Coping‐Together + lay guidance (patient = 100%, caregivers = 91.7%) versus the self‐directed Coping‐Together (patient = 69.6%, caregivers = 68.7%) group. When it came to identifying self‐management skills, 87.8% of patients and 96.4% of caregivers used the booklets, with proportions favoring again Coping‐Together + lay guidance (patients and caregivers = 100%) versus self‐directed Coping‐Together (patients = 78.2%, caregivers = 93.8%).

For the coping plan, 89% of those in the Coping‐Together + lay guidance group prepared one versus 48% for self‐directed Coping‐Together. All those in the Coping‐Together + lay guidance group said they applied their coping plan or self‐management skill, dropping to 58% for those in self‐directed Coping‐Together. Booklets most used were Dealing Stress and Worry, Supporting Each other, and Getting on Top of Symptoms. Coping‐Together was delivered as planned for all dyads with no protocol infringements.

### 3.5 Adherence Coping‐Together + Lay Guidance

On average, participants received 4.3/6 calls (SD = 1.6) and 73.9% of participants received at least 4 calls (patient and/or caregiver present), with an average duration of 25.1 min (SD = 6.4 min). Among the 17 dyads in Stage 1, 9 (52.3%) completed ≥ 4 calls together. Calls were either missed by both members of the dyads or were missed only by the caregiver. Patients spoke almost twice as often in a call as caregivers. 63% of calls were > 20 min; no differences in call length between dyads and patients alone. The guides were found to apply planned procedures and content for 94% and 98% of the calls, respectively.

### 3.6 Adherence Coping‐Together + Lay Guidance + MI

Adherence to the MI calls (*n* = 5 dyads, 1 patient) for non‐responders stepped‐up in Stage 2 exceeded our 75% benchmark. All dyads and patients completed ≥ 4/6 of the calls. Most (83%) participants received the minimum MI dose of 180 min. In terms of each MI process, engage and focus were applied in 91.7% of calls, planning in 88.9% of calls, and evoke in 47.2%. According to the MI interventionist, the lower rate of evoking could be accounted for by a low level of ambivalence present in the participants, who in the context of MI, were more willing to identify challenges and create a change plan.

### 3.7 Helpfulness

In the Coping‐Together + lay guidance group, 89% of participants found the workbook moderately or very helpful versus 63% in the self‐directed Coping‐Together group. For the booklets, 100% of participants gave it the same rating in the Coping‐Together + lay guidance group versus 78% in the self‐directed Coping‐Together group.

### 3.8 Missing Data

Proportion of missing data at baseline was less than 3% and at follow‐up less than 7%.

### 3.9 Clinical Significance

See Table S3: in Supporting Information S1 and Table S4 in supplementary materials for detailed results. At the end of Stage 1, response to Coping‐Together + lay guidance was 57.1% versus 69.6% to Coping‐Together self‐directed. Patients and, less so, caregivers seemed to have benefited more from self‐directed Coping‐Together for anxiety (pooled ES = −0.25 (−0.59; 0.09)). Approximately 10% more patients and caregivers in the self‐directed Coping‐Together group improved their anxiety score by the MCID than in the lay guidance group (see Table S4).

At the end of Stage 2, among patient responders, those who continued with Coping‐Together + lay guidance seemed to report lower anxiety than those who continued with self‐directed Coping‐Together (ES = 0.38 (−0.46; 1.23)), potentially suggesting delayed impact of the guidance. The ES for QOL was non‐significant for this comparison. However, for both anxiety and QOL, all patient non‐responders who were stepped‐up (lay guidance or MI) seemed to have benefited from their new allocation versus non‐responders who continued with their Stage 1 intervention (ES_anxiety_ = 0.26 (−0.89; 1.41); ES_QOL_ = 0.54 (−0.50; 1.57)). These trends were also noted among the MCIDs.

For caregivers, most Stage 2 (12‐week) ESs were < 0.2. The exception is caregiver responders assigned to self‐directed Coping‐Together in Stage 1 who continued with this assignment in Stage 2 seemed to report higher QOL than those in the Coping‐Together + lay guidance in Stages 1 and 2. The ESs for caregiver non‐responders stepped‐up were < 0.2. The MCIDs further suggest that caregiver responders benefited more from self‐directed Coping‐Together and caregiver non‐responders benefited by staying in their Stage 1 assignment.

## 4 Discussion

We examined the feasibility, acceptability, and clinical significance of a time varying self‐management dyadic intervention using a stepped‐care approach. SMART designs are overall quite recent and their application in a dyadic context is even more innovative.

Response to self‐directed Coping‐Together was higher than anticipated at almost 70%, supporting its potential efficacy as a Step 1 within a stepped‐care approach. Others have found that patients [15] and caregivers [37] often prefer to first try self‐directed management of cancer challenges before a guided program. In the present pilot, response was based on DT scores, the most used patient‐reported outcome measure in clinical practice. Recently, the DT was validated for use among caregivers [38], potentially further increasing the feasibility of implementing this response criterion in usual care.

Despite lower response, adherence was higher in the Coping‐Together + lay guidance group. In another study by our team of a self‐directed depression toolkit, adherence and efficacy were higher in the lay guided versus self‐directed group [16]. However, outcome assessment was later, at 3 months post‐intervention, potentially giving more time to participants to practice the learned skills. Our previous research [39] also highlighted that the relationship between adherence and the toolkit's efficacy was not straightforward. Adherence indicators such as writing in the toolkit and number of guidance calls were not associated with depression. Rather, when (early vs. late use) and how (e.g., which sections) the toolkit was used were associated with outcomes. Adherence to Coping‐Together might need to be operationalized through multiple indicators to better understand associations with response/efficacy.

Another consideration for a lower response to lay guidance is that it was too intense or too much work too soon. This might have also led to a higher perceived intervention complexity and then higher attrition [40]. Potentially, some dyads might not have been ready to engage in the self‐management process and guidance “forced” them to do so. In future studies, focusing more on accountability in using Coping‐Together, rather than on applying self‐management, might be less confronting [41]. Another explanation is that participants might have preferred guidance from healthcare professionals, which needs to be further explored in future studies.

Higher attrition among the lay guidance group was also noted in our previous studies [41]. Though, this is the first time that lay guidance was used in a dyadic context [41]. There is an added complexity when trying to have two individuals on the calls and managing dyadic and individual needs. This might have also led to focusing on one member of the dyad more so than the other. Delaying guidance to Stage 2 might give dyads time to peruse Coping‐Together on their own and establish their patterns of “internal” guidance. In a previous study of a dyadic self‐directed online intervention, patients and caregivers were found to “coach” each other in intervention use, in a way that was consistent with their coping patterns [19]. With dyads, “external” guidance might best be suited for Stage 2 among non‐responders to self‐directed Coping‐Together. However, the investment of patient responders in Coping‐Together + lay guidance seemed to have benefited them by the end of Stage 2, a trend not noted for caregivers. These patients might have continued to practice self‐management and saw improvements. This finding does raise questions about when is it best to measure response? Potentially a 2–3 weeks delay post‐intervention might allow participants to integrate what they learned during Stage 1 and lead to a higher response rate to lay guidance.

### 4.1 Clinical Implications

All patient non‐responders benefited from being stepped‐up. Based on the findings of our study, a 3‐step adaptive self‐management support intervention is proposed for further testing: Step 1 –self‐directed Coping‐Together, Step 2—Coping‐Together + lay guidance, and Step 3—Coping‐Together + lay guidance + MI. Howell et al. [2] suggested clinician guidance in Step 2; however, the present study suggests lay guidance in Step 2 followed by clinician support in Step 3 might have a synergistic effect.

The clinical significance patterns were less clear for caregivers but seemed to favor self‐directed Coping‐Together. Caregivers seemed to have benefited more from the flexible, self‐directed format. This is consistent with caregivers' preference for interventions that can be used when and where is convenient [42]. For those in the lay guidance group, needing to attend sessions might have resulted in unwanted pressure [42]. It has also been well documented that caregivers tend to deprioritize their own needs focusing instead on those of the patient [42]. Therefore, whether or not made explicit, shared calls may have defaulted toward the needs of patient resulting in less improvements in caregivers.

### 4.2 Study Limitations and Future Studies

A strength is a rigorous SMART. Limitations include lack of diversity in terms of caregiver relationship and ethnicity. Also, our sample was overall well‐educated for whom a self‐directed approach may be more acceptable. Our sample size was adequate for a pilot SMART; conclusions about efficacy remains tentative. Future studies should consider cost‐effectiveness of the proposed 3‐step approach to self‐management. Future studies could also explore the role of peer guidance within this 3‐step stepped‐care approach. Response was defined by a distal outcome and potentially future studies could consider proximal outcomes (e.g., learning self‐management skills) [20]. Further work is also needed to determine whether the same response variable should be used for patients and their caregivers [20]. Coping‐Together was developed to address top unmet needs of patients with early‐stage breast, prostate, or colorectal cancer and their caregivers. Adaptations for patients with advanced cancer and other high‐need patient and caregivers sub‐groups is ongoing [43] to ensure the intervention includes topics of interest.

## 5 Conclusion

This pilot met most of its a priori benchmarks to justify progressing to a larger trial. Although the potential efficacy of a stepped‐care approach was clearer for patients, caregivers seemed to benefit more from a self‐directed approach. Based on these findings, a 3‐step adaptive self‐management support intervention is proposed for further testing: Step 1 –self‐directed Coping‐Together, Step 2—Coping‐ Together + lay guidance, and Step 3—Coping‐Together + lay guidance + MI. This will allow patients and caregivers to establish “internal” patterns of guidance prior to adding “external” guidance.

## Ethics Statement

All procedures were performed in compliance with relevant laws and institutional guidelines and a multicentre ethics approval was obtained (REB of the MWI IUHSSC ‐ Research Ethics Board of the Center intégré universitaire de santé et de services sociaux de l’Ouest‐de‐l’Île‐de‐Montréal, BIOMEDICAL SUBCOMMITTEE: protocol number: 17‐10, 2017).

## Consent

All participants provided written consent in accordance with approved protocol.

## Conflicts of Interest

The authors declare no conflicts of interest.

## Supporting information

Supporting Information S1

Table S4

## Data Availability

The data that support the findings of this study are available from the corresponding author upon reasonable request.
