# Supplementary material for: Translating Evidence‐Based Self‐Management Interventions Using a Stepped‐Care Approach for Patients With Cancer and Their Caregivers: A Pilot Sequential Multiple Assignment Randomized Trial Design
Source: Psychooncology. 2025 Jan 6;34(1):e70043. doi: 10.1002/pon.70043 (PMC11704335; doi:10.1002/pon.70043)
Supplement: Supplementary file 1 — Supporting Information S1 [file PON-34-e70043-s002.docx]

**Supplementary Materials**

*Table S1. Overview of Coping-Together Booklets*

| **Booklet** | **Description** | **Example challenges** | **Example strategies** |
| --- | --- | --- | --- |
| **Getting on top of symptoms** | Coping with common treatment side effects | 1. Fatigue 2. Pain 3. Nausea | 1. Use a symptom diary, talk to your health care team, and use self-care strategies |
| **Dealing with stress and worry** | Addressing the emotional reactions to diagnosis and treatment | 1. I feel tense, angry and/or stressed 2. I feel worried or uncertain | 1. Use relaxation techniques 2. Use problem-solving techniques |
| **Getting what you need from your health care team** | Working with your medical team, knowing how to ask the right questions, getting and understanding the information you need | 1. We don’t know what questions to ask 2. We leave our appointments feeling we didn’t get what we wanted | 1. Use question checklists 2. Prepare for and play an active role in your medical care |
| **Making your treatment decision** | Considering your options, treatment planning, and adjusting to treatment-related delays | 1. We feel overwhelmed by options 2. We want more of a say in the decision | 1. Understand your options and use decision aids 2. Use assertive communication and consider a second opinion |
| **Supporting each other** | Enhancing your communication and connection to your partner, and adjusting to changes that might arise in your relationship | 1. I just don’t know how to make my partner feel better 2. I am finding it harder to deal with conflict | 1. Use listening skills, body language and empathy, avoid roadblocks to listening well 2. Resolve disagreements in a way that you can be proud of later |
| **Getting the support you need** | Finding appropriate support to address practical, emotional, financial, legal, and informational needs | 1. We need to know what support we have around us 2. We need financial help | 1. Your plan to build a support network in your community 2. Understand what is available, where to find it, and how |

*Table S2. Intervention elements for Guided Coping-Together vs. Coping-Together + MI*

| **Intervention Elements** | **Lay Guidance** | **Motivational Interviewing** |
| --- | --- | --- |
| Sessions | 6 x 1/week | 6 x 1/week |
| Length | 15-20 minutes | 30-45 minutes |
| Duration | 6 weeks | 6 weeks |
| Goal | Provide information and facilitate use of Coping-Together | Strengthen motivation/confidence for adopting self-management skills |
| Approach | Not counselling, lay trained guide | Counselling using MI, implemented by health care professional |
| Content | (a) introduce and provide orientation to Coping-Together; (b) support the identification of a challenge they want to learn to self-manage and create a coping plan; and (c) encourage adherence by setting a SMARTTER goal | (a) engage: confidence in MI practitioner-participant relationship; (b) Focus: conversation the use of self-management; (d) Evoke: elicit participants’ motivation toward the use of self-management; (c) Plan: when participant ready, consolidate commitment to coping plan ^31^ |

*Table S3.* *Effect sizes (ESs) at 6 and 12 weeks (primary outcomes)*

| **Outcomes** | **Effect size [95% CI]** | | | | | |
| --- | --- | --- | --- | --- | --- | --- |
|  | **Unadjusted** | | | **Adjusted for baseline scores** | | |
|  | **Patient** | **Caregiver** | **Pooled** | **Patient** | **Caregiver** | **Pooled** |
| **DT score at** Stage 1^a^ | -0.59  [-1.18; 0.02] | -0.27  [-0.94; 0.39] | -0.49  [-1.01; 0.03] | -0.31  [-0.90; 0.28] | -0.38  [-1.04; 0.29] | -0.38  [-0.85; 0.08] |
| **HADS-anxiety** |  | | | | | |
| Stage 1 ^a^ (1 missing) | -0.66  [-1.26; -0.04] | -0.21  [-0.88; 0.47] | -0.46  [-0.94; -0.01] | -0.36  [-0.96; 0.23] | -0.09  [-0.77; 0.58] | -0.25  [-0.59; 0.09] |
| Stage 2 |  | | | | | |
| 6-week responders at 12 weeks^b^ | -0.40  [-1.24; 0.45] | -0.33  [-1.30; 0.65] | -0.30  [-0.83; 0.23] | 0.38  [-0.46; 1.23] | -0.13  [-1.10; 0.84] | 0.15  [-0.23; 0.54] |
| 6-week non-responders at 12 weeks^c^ | 0.49  [-0.69; 1.64] | 0.66  [-0.84; 2.11] | 0.57  [-0.35; 1.16] | 0.26  [-0.89; 1.41] | 0.07  [-1.36; 1.50] | 0.12  [-0.49; 0.72] |
| **QOL^d^ Stage 2** |  | | | | | |
| 6-week responders at 12 weeks^b^ | -0.18  [-0.97; 0.61] | 0.00  [-0.97; 0.97] | NA | 0.14  [-0.66; 0.94] | -0.36  [-1.34; 0.61] | NA |
| 6-week non-responders at 12 weeks^c^ | 0.81  [-0.26; 1.86] | 0.57  [-0.74; 1.85] | NA | 0.54  [-0.50; 1.57] | -0.16  [-1.43; 1.11] | NA |

Note. All 12-week analyses with completers. NA: Not Applicable. Pooled ES for QOL cannot be done, as different scales used for patients and caregivers. ES with a positive value = Intervention group is more effective. ^a^Coping-Together only (n1=24, n2=21) vs. Coping-Together + guidance (n1=21, n2=15). ^b^Self-directed Coping-Together (n1=14, n2=13) vs. Coping-Together + lay guidance (n1=9, n2=6). ^c^Continued with Stage 1 (n1=5, n2=3) vs. Stepped-up (n1=7, n2=5). ^d^Different QOL measures

## **Cancer Challenges**

## **Coping-Together provides practical information to cope with key cancer challenges**

**Medical** (e.g., participating in treatment decision-making)

**Daily Living Stressors** (e.g., Adjusting normal household routines)

**Social Stressors** (e.g., balancing social and recreational activities)

**Emotional Stressors** (e.g., uncertainty about the future)

**Personal factors** (e.g., beliefs, age, gender, cancer preoccupation)

**Illness-related factors** (e.g., type and stage of cancer)

## **Resources** – Coping-Together will increase internal (e.g., knowledge, health literacy) and external (e.g., social support) resources

## **Cognitive appraisal**

**Primary** (irrelevant, benign-

positive or stress producing) As Coping-Together increases resources to cope, challenges are appraised as less threatening.

**Secondary** (Coping-Together is anticipated to have a positive impact on self-efficacy or evaluation of coping options)

## **Coping** –

## Coping-Together aims to increase repertoire of emotion-and problem-focused coping strategies

Overriding threat

Positive emotion

Positive emotion

**Unfavourable resolution/No resolution**

**Anxiety**

**Quality of life**

**Meaning-based coping**

For stressors that cannot be resolved, Coping-Together sustains meaning-based coping and fosters positive emotions (e.g., reappraisal)

Sustain coping process

*Figure S1.* Coping-Together’s mechanisms of action. Shaded grey box indicate where Coping-Together is expected to have an impact to enhance coping with cancer challenges. Based on frameworks cited in main manscript.
